# Validation of the Tinnitus Acceptance Questionnaire: Japanese Version

**DOI:** 10.3390/audiolres12010006

**Published:** 2022-01-13

**Authors:** So Takabatake, Mariko Takahashi, Kayoko Kabaya, Yoshimasa Sekiya, Kenichi Sekiya, Ikuma Harata, Masaki Kondo, Tatsuo Akechi

**Affiliations:** 1 Department of Psychiatry and Cognitive-Behavioral Medicine, Nagoya City University Graduate School of Medical Sciences, Nagoya 467-8601, Aichi, Japan; so.takabatake@gmail.com (S.T.); takechi@med.nagoya-cu.ac.jp (T.A.); 2 Department of Otolaryngology-Head and Neck Surgery, Aichi Gakuin University School of Dentistry, Nagoya 464-0821, Aichi, Japan; mari-tk@dpc.agu.ac.jp; 3 Department of Otolaryngology-Head and Neck Surgery, Nagoya City University Graduate School of Medical Sciences, Nagoya 467-8601, Aichi, Japan; kabaya@med.nagoya-cu.ac.jp; 4 Sekiya ENT Clinic, Nagoya 460-0004, Aichi, Japan; sekiyayo1@sf.commufa.jp; 5 Department of Otolaryngology-Head and Neck Surgery, Anjo Kosei Hospital, Anjo 446-8602, Aichi, Japan; midoriku82@yahoo.co.jp; 6 Department of Otorhinolaryngology, Kainan Hospital, Yatomi 498-8502, Aichi, Japan; ikumaspecial@hotmail.com

**Keywords:** tinnitus, questionnaire, acceptance, acceptance and commitment therapy, COSMIN, method effects

## Abstract

This study aimed to develop and validate a Japanese version of the Tinnitus Acceptance Questionnaire (TAQ), an instrument that measures the process of intentional acceptance of adverse experiences associated with tinnitus. A total of 125 patients with chronic tinnitus from multiple institutions participated in this study. Participants completed the Japanese versions of the TAQ, Tinnitus Handicap Inventory, Valuing Questionnaire, Acceptance and Action Questionnaire-II, and Hospital Anxiety and Depression Scale. A second TAQ was administered 1–2 weeks later. Because the model fitted poorly in confirmatory factor analysis, exploratory factor analysis was conducted, which yielded a two-factor structure that was divided into forward and reversed item groups. Hypotheses regarding criterion and construct validity were clearly supported. A high Cronbach’s α coefficient value was obtained for the TAQ total score (0.88). The interclass correlation coefficient for test–retest reliability was within the acceptable range (0.95). The results of the exploratory factor analysis were considered to be due to artifacts caused by the characteristics of the Japanese language. The present study confirmed the validity and reliability of the Japanese version of the TAQ in measuring tinnitus-specific receptivity.

## 1. Introduction

Tinnitus is the sensation of hearing a sound without an external source and was reported to be present in 10–15% of adults [1]. The majority of people can adapt to the symptom without distress. However, in people with pre-existing psychological vulnerabilities and/or other concurrent emotional life stressors, tinnitus can trigger high levels of anxiety and depressivity which, interacting with existing psychological stressors, can lead to increasing levels of functional impairment [2]. When tinnitus acts as a form of distress, people experience severe distress, regardless of the tinnitus severity [3]. The effectiveness of Acceptance and Commitment Therapy (ACT), which is a form of cognitive behavioral therapy that focuses on the acceptance of tinnitus-related distress and commitment to living one’s life, has been highlighted in the field of coping with chronic conditions, such as tinnitus [1,4]. The American Academy of Otolaryngology–Head and Neck Surgery guidelines recommended that ACT was an effective treatment for patients with persistent and bothersome tinnitus [5]. In a randomized controlled trial of Acceptance and Commitment Therapy in the treatment of tinnitus, ACT showed a significant effect on sleep disturbance and tinnitus [6].

The aim of ACT is to reduce avoidant behavior and to generate and increase an adaptive behavior that is commensurate with an individual’s values [7]. The process of intentional acceptance of aversive experiences is important in ACT and can be measured by the widely used Acceptance Action Questionnaire (AAQ) [8]. Questionnaires have been developed and used to measure acceptance of specific diseases, such as chronic pain and irritable bowel syndrome [9,10]; for tinnitus, the Tinnitus Acceptance Questionnaire (TAQ) has been developed [11]. The validity and reliability of the TAQ have been verified in the original and German versions [11,12]. In the clinical research for tinnitus treatment, questionnaires such as the Tinnitus Handicap Inventory (THI) and the Tinnitus Functional Index have been used to measure the effectiveness of treatment, measuring the subjective distress level of tinnitus and disability in daily life due to tinnitus in the last week, respectively [13,14]. The TAQ differs from these questionnaires since it measures tinnitus-specific acceptance; ACT for tinnitus promotes tinnitus-specific acceptance, which is thought to reduce the distress caused [6]. In Japan, ACT for chronic tinnitus has been implemented [15]. However, there is no Japanese version of the TAQ. Therefore, this study aimed to develop and test the validity and reliability of a Japanese version of the TAQ.

## 2. Materials and Methods

### 2.1. Procedure and Participants

The research was conducted by multicenter, cross-sectional, psychometric evaluations. Participants were recruited from the otolaryngology departments of one university hospital, one otorhinolaryngology clinic, and two general hospitals (two in the city, one around the town area, and one in the countryside) in Japan from June 2020 to February 2021. The target participants were both outpatients and inpatients with tinnitus that persisted for at least 3 months. All participants were native Japanese speakers who were over the age of 20 years. Neuro-otologists diagnosed all patients according to the Clinical Practice Guidelines for the Diagnosis and Management of Tinnitus 2019 by the Japan Audiological Society [16]. In this study, patients who had difficulty filling in the questionnaire because of physical or mental illness were excluded. Thus, we excluded patients who had difficulty understanding the informed consent about the questionnaire (e.g., dementia, severe schizophrenia) or were considered to have difficulty answering the questions or filling in the profile (e.g., severe mental retardation, difficulty with hand movement due to severe neurodegenerative disease).

All participants filled out five self-administered questionnaires on-site, including sociodemographic variables. Participants who agreed to participate in the retest study were instructed to complete a second TAQ 7–14 days following the first session and were asked to fill in the exact completion date. The retest questionnaire was returned by mail or by hand.

This study was based on classical test theory according to the COnsensus-based Standards for the selection of health Measurement INstruments (COSMIN) study design checklist, which is recommended when designing studies to evaluate the measurement properties of existing patient-reported outcome measures [17].

### 2.2. Measurements

#### 2.2.1. TAQ

The TAQ is a 12-item self-reported questionnaire that measures acceptance associated with tinnitus (Table 1) [11]. It uses a seven-point Likert scale, as follows: 0 (never true), 1 (very rarely true), 2 (seldom true), 3 (sometimes true), 4 (often true), 5 (almost always), and 6 (always true). Eight items are reversed items (inverted scales), and the total score ranges from 0 to 72, with higher values indicating higher levels of acceptance. The TAQ has a two-factor structure: “Activity engagement” and “Tinnitus suppression”. “Activity engagement” was associated with behavioral activation and evaluated the presence or extent that a person continued activities of daily living, regardless of tinnitus [11,12]. “Tinnitus suppression” measured attempts to control tinnitus-related cognitions and emotions, and thus represented a measure of experiential avoidance [11,12]. The validity and reliability of the original and German versions of the TAQ have been confirmed [11,12].

#### 2.2.2. Tinnitus Handicap Inventory

The Tinnitus Handicap Inventory (THI) is a 25-item self-reported questionnaire that has been widely used in tinnitus clinical practice and research to assess for distress and handicap associated with tinnitus [13]. The THI comprised items on three subscales as follows: (1) functional (disability affecting daily life); (2) emotional (anxiety and mental stress); and (3) catastrophic (represented the patient’s hopelessness, loss of control, and inability to cope with the problem). Three answers are provided for each question: 4 (yes), 2 (sometimes), and 0 (no). Higher total scores indicated severe handicap with tinnitus. The validity and reliability of the Japanese version have been confirmed [18].

#### 2.2.3. Valuing Questionnaire

The Valuing Questionnaire (VQ) is a 10-item self-reported questionnaire that measures behavior consistent with the ACT’s core process “value” [19]. Each item in the VQ was rated on a seven-point scale from 0 (not at all true) to 6 (completely true); a score was calculated for each of the two factors, progress (VQ-P) and obstruction (VQ-O). VQ-P measured the extent to which the participants were aware of what was personally important and their perseverance toward this. VQ-O measured the extent to which living in line with the values was disrupted by avoiding unwanted experiences and distraction from values, either by neglect or by focusing on other psychological experiences. The validity and reliability of the Japanese version have been confirmed [20].

#### 2.2.4. Acceptance and Action Questionnaire-II

The Acceptance and Action Questionnaire-II (AAQ-II) is a seven-item self-reported questionnaire that measures psychological flexibility [8]. The items were rated on a scale of 1 (never true) to 7 (always true). Higher scores indicated higher levels of psychological inflexibility as well as higher levels of experiential avoidance, which is the attempt to avoid contact with unwanted internal experiences, such as emotions, thoughts, and memories. The validity and reliability of the Japanese version have been confirmed [21].

#### 2.2.5. Hospital Anxiety and Depression Scale

The Hospital Anxiety and Depression Scale (HADS) is a 14-item self-reported questionnaire that measures a patient’s general anxiety and depression without the influence of various physical symptoms [22]. The scale comprised two subscales, including the anxiety subscale (HADS-A) and the depression subscale (HADS-D), both of which comprised seven items that were evaluated on a four-point scale. HADS-A and HADS-D scores of ≥8 reflected the presence of anxiety disorder and depression, respectively [22]. The validity and reliability of both the original and Japanese versions of the HADS have been confirmed [23,24].

### 2.3. Translation and Cross-Cultural Adaptation

After receiving permission from the developer of the original version to create a Japanese version of the TAQ, subsequent steps were taken to translate and back-translate the TAQ. Two native Japanese speakers with sufficient linguistic ability in both English and Japanese each translated the original version of the TAQ into Japanese; one was a professional translator with no prior knowledge on the TAQ, and the other was an otolaryngologist who specialized in tinnitus and had prior knowledge on the TAQ. Another two translators who specialized in psychiatric clinical research and had sufficient linguistic skills in both English and Japanese reviewed the forward-translated Japanese version using a Japanese methodology and resolved discrepancies in the Japanese translation through discussion. An otolaryngologist and a native English-speaking professional translator who had no prior knowledge on the TAQ and had adequate language skills in both English and Japanese back-translated the provisional Japanese version into English. Furthermore, authors of the original version of the TAQ verified the meaning and conceptual equalities between the original and back-translated English versions of the TAQ.

To confirm cross-cultural adaptation, a preliminary Japanese version was tested on nine Japanese patients with chronic tinnitus. None of the patients reported difficulty in understanding the phrasing. The final Japanese version of the TAQ was then finalized.

### 2.4. Data Screening

We excluded cases with outliers and missing values. Each TAQ item was checked to see if it was normally distributed. According to the guideline, if the skewness was ≤2.0 and the kurtosis was ≤7.0, the item can be regarded as a normal distribution [25]. The floor and ceiling effects were considered to be present when ≥15% of the participants got the lowest or highest score [26].

### 2.5. Structural Validity

We performed a confirmatory factor analysis (CFA) based on the assumption of a two-factor model, which corresponded to the factor “activity engagement” and “tinnitus suppression.” The maximum likelihood method was used when each item of the TAQ followed a normal distribution, and the generalized least squares method was used otherwise. Standardized root mean square residual (SRMR), comparative fit index (CFI), and root mean square error of approximation (RMSEA) were used as model fit criteria. A good fit was indicated by an SRMR of ≤0.08, CFI of ≥0.95, and RMSEA of ≤0.05 [25].

When the results of the CFA indicated inadequate fit of the model, an exploratory factor analysis (EFA) was conducted. To assess the goodness of fit of the data for the EFA, the Kaiser–Meyer–Olkin measure of sampling adequacy (KMO) and Bartlett’s test of sphericity were calculated. These values should exceed the recommended minimum of 0.60. To clarify the optimal number of factors, we used both the Kaiser criterion (i.e., the “eigenvalue greater than one” rule) and the scree plot. Assuming the presence of correlation among the factors, we used the oblique Promax rotation.

With the factor loadings becoming negative when reversed items are included in the scale, in this study all reversed items were rescored before factor analysis was conducted.

### 2.6. Other Psychological Characteristics

For criterion and construct validity, we assessed univariate relationships using Pearson correlation coefficients for six hypotheses. For the strength of the relationship, we followed some suggestions that correlation coefficients of ±0.50, ±0.30, and ±0.10 should be considered as large, moderate, and weak relationships, respectively [27,28]. The six hypotheses were as follows:

**Hypothesis** **1.** *Acceptance for tinnitus-related symptoms was expected to be related to the handicaps associated with tinnitus, and the TAQ total score was expected to have a high negative correlation (r < −0.50) with the THI total score.*

**Hypothesis** **2.** *Acceptance for tinnitus-related symptoms was expected to be related to the acceptance of unpleasant private events, and the TAQ total score was expected to have a high negative correlation (r < −0.50) with the AAQ-II total score.*

**Hypothesis** **3.** *Acceptance for tinnitus-related symptoms was expected to be related to the general level of anxiety, and the TAQ total score was expected to have a high negative correlation (r < −0.50) with the HADS-A score.*

**Hypothesis** **4.** *Acceptance for tinnitus-related symptoms was expected to be related to the depressive symptoms, and the TAQ total score was expected to have a high negative correlation (r < −0.50) with the HADS-D score.*

**Hypothesis** **5.** *Acceptance for tinnitus-related symptoms was expected to be related to the extent to which people fulfill their values, regardless of psychological distress, and the activity engagement score on the TAQ was expected to have a high positive correlation (r > 0.50) with the VQ-P score.*

**Hypothesis** **6.** *Acceptance for tinnitus-related symptoms was expected to be related to the extent to which empiric avoidance interfered with value-based behavior, and the tinnitus suppression score on the TAQ was expected to have a high negative correlation (r < −0.50) with the VQ-O score.*

We calculated the Cronbach’s α coefficient for the TAQ total score; a Cronbach’s α coefficient of 0.70–0.95 was considered good for internal consistency [26]. For test–retest reliability of the TAQ total score, the intraclass correlation coefficient (ICC) was calculated as a measure of absolute concordance for a single measurement under a two-way random model; an ICC of 0.70 was suggested as the minimum reliability standard [26].

### 2.7. Sample Size, Statistical Analysis, and Ethics

The sample size of this study was set at 120 because this number met the COSMIN guideline criteria for “very good” (7 times the number of items and at least 100) [17]. The target number of respondents for the retest sample was >50 [17]. CFA was conducted using SPSS Amos for Windows ver. 27 (IBM, Chicago, IL, USA). Other analyses were conducted using SPSS Statistics for Windows ver. 27 (IBM, Chicago, IL, USA). A two-tailed *p* value of <0.05 was considered significant.

This study was approved by the ethics committee of Nagoya City University Graduate School of Medical Sciences and by similar ethical review committees of all other research sites, in accordance with the guidelines provided by the Helsinki Declaration. Written informed consent was obtained from all participants.

## 3. Results

### 3.1. Data Quality

A total of 144 patients filled out the questionnaires. Excluding 13 cases with missing values and six cases with outliers, 125 participants were included in the study (86.8%). We found no methodical differences between the total and the excluded samples. The questionnaire scores and sociodemographic data were not significantly different across all outpatient recruitment sites (sex: χ^2^(3) = 1.62, *p* = 0.656; marital status: χ^2^(3) = 6.39, *p* = 0.094; educational status: χ^2^(3) = 1.81, *p* = 0.614; occupational status: χ^2^(9) = 12.95, *p* = 0.165). The retest sample comprised 78 participants. The demographic features and the clinical condition of the total and retest samples are shown in Table 2. We did not find any systematic differences in total scores and sociodemographics between the retest sample and original sample. As shown in Table 1, there was no floor or ceiling effect in the subscale and total scores.

### 3.2. Structural Validity

**Figure 1:**
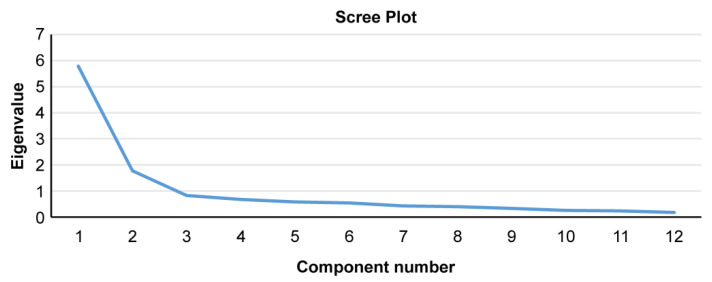
Scree plot of the TAQ items.

To examine whether the Japanese version of the TAQ would have the same two-factor structure as did the original version, a CFA was conducted after processing the reversed items. Model fit indices for CFA considering the assumed two-factor structure indicated a poor model fit; the SRMR, CFI, and RMSEA were 0.1118, 0.770, and 0.165 (90% confidence interval: 0.144–0.187). Therefore, after processing the reversed items, EFA was performed. KMO was 0.88 and Bartlett’s test of sphericity was significant (*p* < 0.001), confirming that the data were suitable for EFA. The 12 items of the TAQ were subjected to factor analysis using the maximum likelihood method. Because both the Kaiser criterion and the scree plot indicated two-factor results, the two-factor solution was adopted (Figure 1). Assuming two factors, we conducted factor analysis with the maximum likelihood method and Promax rotation. The final factor patterns, interfactor correlations, and commonalities after the Promax rotation are shown in Table 3. The patterns of the two-factor subscale were different from those of the original version. Except for item 7, which had the smallest commonality, the two factors in this study consisted of the forward and reversed item groups.

### 3.3. Other Psychological Characteristics

Based on skewness of <2.0, kurtosis of <7.0, and the distribution graph, the total and subscale scores of the four questionnaires were regarded as a normal distribution. Therefore, the Pearson correlation coefficient was used to evaluate the criterion and construct validity. The correlation matrix for the criterion and construct validity clearly supported hypotheses 1, 2, 3, and 4 (Table 4). Hypotheses 5 and 6 could not be tested because the two-factor structure of the TAQ was not confirmed, unlike that in the original version. Therefore, the correlation coefficients of the total TAQ scores with the VQ-P and VQ-O were calculated; a moderate correlation between the total TAQ scores and the VQ-P and VQ-O was confirmed. For internal consistency, the Cronbach’s α coefficient of the TAQ was 0.88 and considered good. In the corrected item–total correlation, item 7 was negative. If item 7 was deleted, the α coefficient was 0.91. The ICC for test–retest reliability was measured in 78 participants who completed the retest. The ICC value in the retest sample (0.95) was acceptable.

## 4. Discussion

The development of a Japanese version of the TAQ was necessary to assess tinnitus-specific acceptance when administering ACT for tinnitus in Japan. To the best of our knowledge, this was the first study that developed and tested the validity and reliability of a Japanese version of the TAQ in accordance with the latest COSMIN guidelines [17]. We confirmed that, except for the factor structure, the Japanese version of the TAQ was a reliable and valid questionnaire.

After rescoring the reversed items, a Promax rotation using the maximum likelihood method was performed, and two factors were extracted. No item had a factor loading <0.5. The two-factor structure consisted of reversed and forward (non-reversed) items except for item 7, which had a commonality <0.3. In the measurement methods that had a mixture of positive and negative questions, the factors may have been split by artifacts that were not related with the essential basic dimensions, such artifacts that are known as method effects [29,30]. In one study on the 10-item version of the AAQ-II, which is a scale that measures acceptance and the TAQ, a two-factor structure that comprised reversed and non-reversed items was confirmed, thereby, showing the influence of the method effects [8]. The factor analysis of the present study resulted in a two-factor structure that comprised reversed and forward (non-reversed) items, and this structure did not correspond to those in previous studies [11,12]. Therefore, when the Japanese version of the TAQ is used, the two subscales of the original version of the TAQ should not be assessed.

We believe that a possible reason for the difference in the response patterns between the reversed and non-reversed items in the Japanese version was the influence of ambiguous expressions peculiar to the Japanese language on the patients’ responses. The ambiguity of the Japanese language creates a tendency to emphasize more contextual information than the content of the sender’s utterance, and the receiver would understand the linguistic content from the contextual information based on independent judgment [31]. This fact suggested that, depending on the patient’s context, ambiguity in Japanese may distort the answer to a question. In other words, in this study, a negative contextual information on tinnitus may have distorted the patients’ attitudes toward answering questions about the internal construct of acceptance. Given the characteristics of the Japanese language, it seemed better to consider the reversed items in the translation. Therefore, similar to the Japanese version of the AAQ-II that is widely used in Japan, we suggest against the creation of reversed items in the Japanese version of the TAQ; this will enable the patients to answer questions without being confused by the ambiguity of the Japanese language.

In the assessment of the criterion and construct validity, a relatively strong correlation was shown between the TAQ and the AAQ-II. The results showed an even higher correlation, compared with that for the original version of the TAQ. In this first study that evaluated correlations with multiple variables other than the AAQ-II, there were significant correlations between the TAQ and measures of anxiety and depression, as hypothesized. These results indicated that the Japanese version of the TAQ was as appropriate as its original version for assessing tinnitus-specific acceptance. For reliability, the Japanese version of the TAQ showed good internal consistency, as did the original and German versions, and the ICC in the retests was sufficiently high to confirm reliability [26].

The strength that enhanced the clinical significance of this study was its robust design following the latest COSMIN guidelines [17]. In addition, our subjects seemed to represent the typical sample of the target population who answered the TAQ, because they were recruited on the basis of diagnosis of neuro-otology specialists at several institutions in various areas. The subjects’ clinical status and demographic characteristics appeared to be close to the actual practice of otolaryngology. The limitation of this study was that it did not assess the responsiveness or interpretability of the Japanese version of the TAQ. Further studies are needed to investigate these characteristics. In this study, patients with conditions that made it difficult for them to complete the self-administered questionnaire due to physical or mental illness were excluded; therefore, patients with more serious emotional distress may not have been included. It is unclear what percentage of such patients were present in this study, as the forms were not distributed to them. However, since such patients are not subjected to the TAQ even in actual clinical practice, it would have little impact on the purpose of this study, which is to verify the reliability and validity of the TAQ.

## 5. Conclusions

In conclusion, this study confirmed the validity and reliability of the Japanese version of the TAQ, according to the latest COSMIN guidelines [17]. The factor analysis in this study resulted in a two-factor structure divided into reversed and forward (non-reversed) items. Considering the characteristics of the Japanese language, it would be better to not create reversal items for the Japanese version of the TAQ. Because the two-factor structure obtained in this study differed from the subscales of the original version, the two subscales of the original version of the TAQ should not be assessed.

## Figures and Tables

**Table 1:** Overall distribution profile of the Tinnitus Acceptance Questionnaire (*n* = 125).

Item		Mean ± SD	Skewness	Kurtosis	Lowest (%)	Highest (%)
**Activity engagement subscale score**		35.22 ± 10.62	−0.17	−0.63	0.0	3.2
1	I am leading a full life, even though I have chronic tinnitus.	4.74 ± 1.21	−1.08	0.45	0.0	27.2
2 *	My chronic tinnitus has led me to decrease my engagement in former activities.	3.21 ± 1.57	0.07	−0.61	4.0	9.6
3	My life is going well, even though I have chronic tinnitus.	4.53 ± 1.29	−1.02	0.69	0.8	21.6
5	Despite tinnitus, I can draw up and stick to a certain course in my life.	4.26 ± 1.59	−1.08	0.52	4.8	20.0
6	When my tinnitus increases I can still take care of my responsibilities.	4.33 ± 1.48	−0.98	0.47	2.4	21.6
8 *	My tinnitus leads me to avoid certain situations.	3.53 ± 1.92	−0.08	−1.26	4.8	25.6
9 *	My tinnitus changes me as a person.	3.42 ± 1.83	−0.11	−1.07	4.8	18.4
10 *	I have to struggle to get things done when I have tinnitus.	3.74 ± 1.79	−0.27	−0.94	3.2	25.6
12 *	I spend a lot of time thinking how things would be for me, without chronic tinnitus.	3.49 ± 1.80	−0.30	−0.80	7.2	17.6
**Tinnitus suppression subscale score**		7.89 ± 3.50	0.30	0.38	1.6	1.6
4 *	It is necessary for me to control my negative thoughts and feelings concerning tinnitus.	2.96 ± 1.63	0.11	−0.50	7.2	8.8
7 *	I will be in better control of my life if I can control my negative thoughts about tinnitus.	1.74 ± 1.57	1.01	0.38	21.6	3.2
11 *	I strive to suppress aversive thoughts and feelings related to tinnitus.	3.19 ± 1.74	0.05	−0.88	5.6	13.6
**Total score**		43.11 ± 12.79	0.0	−0.51	0.0	1.6

SD: Standard Deviation. * Items **2, 4, 7, 8, 9, 10**, **11**, **and 12** are reversed items.

**Table 2:** Clinical condition and demographic features of the patients included in this study.

	Total Sample (*n* = 125)		Retest Sample (*n* = 78)	
	**N**	**(%)**	***n***	**(%)**
**Male**	66	(52.8)	40	(51.3)
Age (years) *	60.7	±13.2	60.3	±12.4
**Marital status**				
Married	96	(76.8)	64	(82.1)
Unmarried/Divorced/Widowed	29	(23.2)	14	(17.9)
**Educational status**				
Junior high school/High school	40	(32.0)	20	(25.6)
Junior college/University	85	(68.0)	58	(74.4)
**Occupational status**				
Full-time worker	45	(36.0)	27	(34.6)
Part-time worker	24	(19.2)	16	(20.5)
Housewife	23	(18.4)	15	(19.2)
Student	0	(0.0)	0	(0.0)
Others	33	(26.4)	20	(25.6)
**Tinnitus type**				
Binaural tinnitus	65	(52.0)	43	(55.1)
Right tinnitus	21	(16.8)	13	(16.7)
Left tinnitus	35	(28.0)	21	(26.9)
Noises in the head	4	(3.2)	1	(1.3)
**Hearing loss**	91	(72.8)	54	(69.2)
**Diagnosis**				
Sudden deafness	16	(12.8)	12	(15.4)
Presbycusis	36	(28.8)	21	(26.9)
Otosclerosis	3	(2.4)	2	(2.6)
Chronic otitis media	1	(0.8)	1	(1.3)
Tinnitus without hearing loss	29	(23.2)	22	(28.2)
Acoustic trauma	5	(4.0)	2	(2.6)
Acute low-tone sensorineural hearing loss	1	(0.8)	1	(1.3)
Head injuries	1	(0.8)	0	(0.0)
Ménière’s disease	8	(6.4)	3	(3.8)
Other sensorineural hearing loss ^†^	9	(7.5)	5	(6.4)
Unexplained	16	(12.8)	9	(11.5)
Disease duration (months) *	114.7	±125.2	122.6	±134.2
**Mental status**				
Clinical anxiety (HADS-A ≥ 8)	35	(28.0)	18	(23.1)
Clinical depression (HADS-D ≥ 8)	41	(32.8)	25	(32.1)

* Mean ± SD. † Sensorineural hearing loss which is not defined by the diagnostic guideline of the Japan Audiological Society; examples are unexplained sensorineural hearing loss and acoustic neuroma.

**Table 3:** Coefficients of factor pattern and matrix, interfactor correlation, and commonality in the exploratory factor analysis.

		Pattern Coefficient		
	**Outline of Item**	**F1**	**F2**	*h^2^*
**10**	I have to struggle to get things done when I have tinnitus.	**0.902**	−0.054	0.761
**11**	I strive to suppress aversive thoughts and feelings related to tinnitus.	**0.883**	−0.165	0.640
**9**	My tinnitus changes me as a person.	**0.778**	−0.063	0.553
**12**	I spend a lot of time thinking how things would be for me, without chronic tinnitus.	**0.707**	0.079	0.570
**4**	It is necessary for me to control my negative thoughts and feelings concerning tinnitus.	**0.703**	0.007	0.500
**8**	My tinnitus leads me to avoid certain situations.	**0.683**	0.014	0.478
**2**	My chronic tinnitus has led me to decrease my engagement in former activities.	**0.590**	0.129	0.452
5	Despite tinnitus, I can draw up and stick to a certain course in my life.	0.045	**0.869**	0.801
1	I am leading a full life, even though I have chronic tinnitus.	0.127	**0.682**	0.580
3	My life is going well, even though I have chronic tinnitus.	0.206	**0.665**	0.642
6	When my tinnitus increases I can still take care of my responsibilities.	0.046	**0.653**	0.463
**7**	I will be in better control of my life if I can control my negative thoughts about tinnitus.	0.310	**−0.609**	0.252
	**Interfactor correlation**	F1	F2	
	F1		0.571	
	F2	0.571		

Exploratory factor analysis used the maximum likelihood method, Promax rotation. Factor pattern coefficients of 0.500 or more are in bold. Items **2, 4, 7, 8, 9, 10**, **11**, **and 12** are reversed items. *h*^2^: commonality.

**Table 4:** Criterion and construct validity: correlation coefficients between variables.

	TAQ	THI	VQ-P	VQ-O	AAQ	HADS-A	HADS-D
TAQ							
THI	**−0.810**						
VQ-P	0.320	−0.386					
VQ-O	−0.369	0.452	−0.261				
AAQ-II	**−0.596**	0.711	−0.434	0.597			
HADS-A	**−0.546**	0.620	−0.382	0.429	0.670		
HADS-D	**−0.585**	0.661	−0.591	0.435	0.676	0.732	

All correlations are significant (*p* < 0.01). Numbers in bold indicate the relationships described in the prior hypotheses. TAQ: Tinnitus Acceptance Questionnaire; THI: Tinnitus Handicap Inventory; AAQ-II: Acceptance and Action Questionnaire-II; VQ-P/O: Valuing Questionnaire-Progress/Obstruction; HADS-A/D: Hospital Anxiety and Depression Scale-Anxiety subscale/Depression subscale.

## Data Availability

Not applicable.
